# Body mass index mediates the association between children's dietary inflammatory index and obstructive sleep apnea-hypopnea syndrome in children: a cross-sectional study

**DOI:** 10.3389/fpubh.2026.1772032

**Published:** 2026-06-05

**Authors:** Yi Wen, Jinglan Chen, Xia Yang, Shiqi Xie, Jianrong Zhou, Xiaozhu Zhang

**Affiliations:** 1 School of Nursing, Chongqing Medical University, Chongqing, China; 2 Department of Eye and Otorhinolaryngology, Women and Children's Hospital of Chongqing Medical University, Chongqing, China; 3 School of Social Development and Public Policy, Fudan University, Shanghai, China

**Keywords:** body mass index, children's dietary inflammatory index, cross-sectional study, mediating effect, pediatric obstructive sleep apnea-hypopnea syndrome

## Abstract

**Background:**

The Children's Dietary Inflammatory Index (C-DII) is associated with elevated body mass index (BMI), a major risk factor for pediatric obstructive sleep apnea-hypopnea syndrome (OSAHS). However, the interrelationships between pro-inflammatory diets, BMI, and OSAHS severity in children remain unclear. This study aimed to examine the association between C-DII and OSAHS severity and assess whether BMI statistically explained the association.

**Methods:**

This cross-sectional study involved 297 children diagnosed with OSAHS. Dietary intake was assessed using a 3-day 24-h dietary recall (including two weekdays and one weekend) to calculate the C-DII scores. All participants underwent polysomnography to determine the obstructive apnea-hypopnea index (OAHI) as an indicator of OSAHS severity. Multiple linear regression analyses evaluated the association between C-DII tertiles (T1 as reference compared to T2 and T3) and OAHI, adjusting for key sociodemographic and clinical covariates. A mediation analysis using the PROCESS macro quantified the proportion of the association statistically attributable to BMI.

**Results:**

After covariate adjustment, the highest C-DII tertile (T3, pro-inflammatory diet) had a significantly higher OAHI vs. the lowest tertile (T1) (β = 2.04, 95% CI: 0.07–4.01, *p* = 0.042). This difference is equivalent to an increase of approximately two obstructive events per hour. The association attenuated and lost significance after adding BMI to the model (β = 1.58, 95% CI: −0.37–3.54, *p* = 0.112). BMI remained strongly associated with OAHI (β = 0.53, *p* = 0.001). Mediation analysis indicated that BMI statistically accounted for 20.18% of the total observed association between C-DII on OAHI.

**Conclusions:**

A pro-inflammatory diet (higher C-DII score) is associated with more severe pediatric OSAHS. This association was largely statistically explained by BMI. Causality cannot be inferred from this cross-sectional study. These findings support integrating dietary and weight strategies in pediatric OSAHS care. Nonetheless, longitudinal studies are needed to clarify diet-specific effects beyond weight control.

## 1 Introduction

Obstructive Sleep Apnea-Hypopnea Syndrome (OSAHS) is a common sleep-related breathing disorder characterized by recurrent episodes of partial or complete upper airway obstruction during sleep, leading to chronic intermittent hypoxia and sleep fragmentation (1, 2). It affects approximately 1 to 5% of children worldwide and constitutes a significant public health challenge (3). The consequences of pediatric OSAHS extend beyond disrupted sleep and encompass neurocognitive deficits, impaired growth, cardiovascular and metabolic complications, and adverse effects on mental health and behavioral development (4–6). The substantial burden imposed on affected children, their families, and healthcare systems underscores the urgent need for effective prevention and management strategies (7, 8).

The pathogenesis of pediatric OSAHS is multifactorial, involving anatomical fac-tors such as adenotonsillar hypertrophy, craniofacial abnormalities, and various socio-demographic and environmental influences (2, 9, 10). Among these, obesity has been unequivocally identified as a major independent risk factor (11–13). Excess neck fat deposition can directly compress the upper airway (14, 15) and this effect can synergistically worsen airway collapse when combined with adenotonsillar hypertrophy (16, 17). Furthermore, abdominal obesity can reduce chest wall compliance thereby further compromising ventilation during sleep (18, 19). Evidence indicates a strong positive correlation between Body Mass Index (BMI) and OSAHS prevalence with rates being significantly higher in children with overweight or obesity compared to their peers without overweight (20). Critically, the co-occurrence of obesity and OSAHS is linked to exacerbated systemic chronic low-grade inflammation which represents a shared pathophysiological mechanism that may amplify metabolic dysfunction and cardiovascular risk (21, 22). Obesity is characterized by a pro-inflammatory state involving the release of cytokines (23) and this state may be further amplified by the intermittent hypoxia inherent to OSAHS, creating a vicious cycle of inflammation (24).

In recent years, dietary patterns have emerged as pivotal, modifiable lifestyle fac-tors capable of modulating systemic inflammation. The Dietary Inflammatory Index (DII) is a well-validated tool designed to quantify the overall inflammatory potential of an individual's diet based on its content of various nutrients and food parameters (25). Positive scores reflect a pro-inflammatory potential and negative scores reflect an anti-inflammatory potential (25). Its adaptation for pediatric populations, the Children's Dietary Inflammatory Index (C-DII), has been developed and validated to assess dietary inflammation in children and adolescents (26). A growing body of evidence consistently demonstrates that higher C-DII scores which reflect a more pro-inflammatory diet are significantly associated with increased BMI and a greater likelihood of having obesity in children (27–29). A pro-inflammatory diet, often high in saturated fats and refined carbohydrates and low in fiber, has been hypothesized to promote weight gain through mechanisms that include enhancing oxidative stress and disrupting energy-regulating hormones like leptin and adiponectin (30, 31).

OSAHS itself is recognized as a chronic inflammatory disorder in which recurrent hypoxia-reoxygenation cycles activate inflammatory pathways leading to the upregulation of cytokines such as interleukin-6 (IL-6), tumor necrosis factor-α (TNF-α), and interleukin-8 (IL-8) (32, 33). Unhealthy, pro-inflammatory diets have been linked to various adverse sleep outcomes including poor sleep quality (34), and anti-inflammatory dietary interventions have shown promise in improving sleep parameters (35). In adults, higher DII scores have been associated with a higher apnea-hypopnea index (AHI) and worse daytime sleepiness in OSAHS patients (36). Notably, when analyses are stratified by BMI the association between a pro-inflammatory diet and OSAHS appears more pronounced in individuals with obesity. This observation suggests that BMI may lie on the pathway linking diet to OSAHS severity (37).

However, the etiology, dietary habits, and physiological context of pediatric OSAHS differ fundamentally from those in adults, preventing the direct extrapolation of adult findings to children. To date, evidence exploring the specific association be-tween the C-DII and OSAHS severity in pediatric populations is scarce. Furthermore, although obesity is a well-established risk factor for OSAHS and diet is a determinant of obesity, the statistical interrelationship among these three variables in children remains uninvestigated. Therefore, we designed this cross-sectional study to address this critical knowledge gap. Based on the biological rationale that a pro-inflammatory diet may promote obesity and that obesity, in turn, increases OSAHS risk, we conceptualized BMI as a potential statistical intermediary in the observed associations linking C-DII to OSAHS severity. We hypothesized that: (1) a higher C-DII score (indicating a more pro-inflammatory diet) is associated with greater disease severity as measured by the Obstructive Apnea-Hypopnea Index (OAHI) in children with OSAHS, and (2) the observed association between C-DII and OAHI is statistically accounted for in part by BMI. These findings may provide novel theoretical support for integrating dietary and weight management strategies into the comprehensive care of pediatric OSAHS.

## 2 Materials and methods

### 2.1 Study design

This cross-sectional study was conducted at the Department of Otolaryngology in a tertiary hospital in Chongqing, China, from December 2024 to May 2025. Participants were recruited using a convenience sampling method. The study protocol was approved by the Ethics Committee of the First Affiliated Hospital of Chongqing Medical University (Approval Number: 2024-615-01). The study was conducted in full accordance with the ethical principles of the Declaration of Helsinki. The reporting of this study followed the Strengthening the Reporting of Observational Studies in Epidemiology (STROBE) guidelines (38) (see Supplementary Table 1). Written informed consent was obtained from the guardians of all participating children, and assent was obtained from the children where appropriate.

### 2.2 Participants

**Figure 1:**
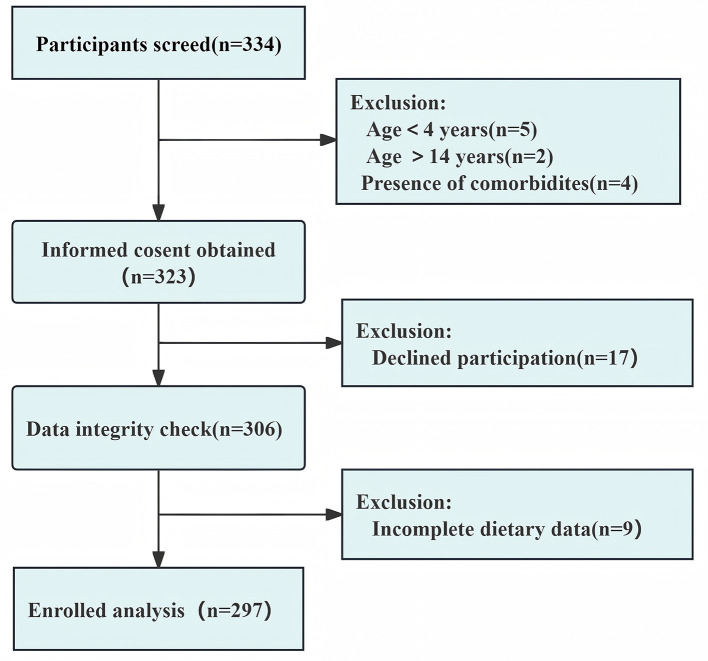
Flowchart of the study participants.

The participant flow is summarized in Figure 1. Initially, 334 children were screened for eligibility.

The inclusion criteria for children were: (1) a diagnosis of OSAHS, defined as an Obstructive Apnea-Hypopnea Index (OAHI) > 1 event/h on polysomnography (PSG), accompanied by symptoms such as snoring, mouth breathing, or witnessed apnea; (2) aged between 4 and 14 years; and (3) provision of informed consent by a guardian.

Exclusion criteria included: (1) a history of adenotonsillectomy; (2) congenital craniofacial malformations, neurological disorders, or chronic inflammatory diseases (e.g., asthma, autoimmune diseases); and (3) severe cardiopulmonary, hepatic, renal, or neoplastic diseases.

Primary caregivers, who provided the questionnaire and dietary data, were required to be aged 18 years or older, live with the child, and have no cognitive impairments or psychiatric disorders.

After screening, 37 children were excluded (seven for age, four for comorbidities, 17 declined, and nine with implausible or erroneous dietary intake records that could not be verified during the interview). In the final analytical sample of 297 participants, no data were missing for any variable. All questionnaires and clinical measurements were completed during face-to-face encounters, and any discrepancies in dietary recall were clarified with the caregiver immediately, ensuring data completeness.

### 2.3 Sample size

An *a priori* power analysis was conducted using G^*^Power software to estimate the sample size required for testing the significance of the incremental variance explained (Δ*R*^2^) by the proposed mediation path (via BMI) in a multiple linear regression model. As this is the first study to investigate BMI as a statistical intermediary between the C-DII and OSAHS severity in children, there were no direct estimates of the anticipated effect size (*f*^2^) for the mediation pathway. Therefore, A conservative, small-to-medium effect size (*f*^2^ = 0.15) was selected to ensure adequate power to detect a clinically meaningful mediation effect while not being overly optimistic. With this effect size, a significance level (α) of 0.05, a power of 80%, and accounting for a total of 14 predictor variables in the full model (including covariates), the analysis indicated a minimum required sample size of 155 participants. Our final sample of 297 participants exceeds this requirement.

### 2.4 Sociodemographic and clinical outcome measures

Trained investigators collected participants' sociodemographic information, including age, sex, caregivers' employment status, and parental education level. Anthropometric indices included body height and weight. Body mass index (BMI) was calculated as weight in kilograms divided by the square of height in meters (kg/m^2^). Participants were classified as normal weight, overweight, or obese according to the age- and sex-specific BMI cut-off values and BMI percentile criteria for Chinese children (39).

Clinical outcome measures included the obstructive apnea-hypopnea index (OAHI) obtained through polysomnography (PSG). An OAHI > 1 event/h was used as the diagnostic criterion for OSAHS. The severity grading of OAHI was consistent with the pediatric scoring criteria of the American Academy of Sleep Medicine (AASM) (40). Participants were further categorized by OSAHS severity according to the following OAHI thresholds: mild (1 < OAHI ≤ 5 events/h), moderate (5 < OAHI ≤ 10 events/h), and severe (OAHI > 10 events/h). Additionally, airway obstruction severity was evaluated based on the adenoidectomy/nasopharyngeal ratio (A/N ratio) in lateral nasopharyngeal radiographs. A/N ratio classification criteria: normal (A/N ≤ 0.60), physiological hypertrophy (A/N 0.61–0.70), and pathological hypertrophy (A/N ≥ 0.71) (41).

### 2.5 Dietary assessment

Trained research investigators conducted a 3-day 24-h dietary recall to interview participants and their caregivers to assess the types and amounts of foods consumed. Participants and their caregivers were asked to thoroughly recall and describe all meals consumed over three non-consecutive days. This included two weekdays and one weekend day to account for within-week variation in dietary intake. Detailed information was collected on food names, portion sizes, and eating occasions, such as breakfast, lunch, dinner, and snacks. Standardized food atlases, measuring tools (such as spoons and cups), and portion-size photographs were used to assist caregivers in recalling food intake. Subsequently, daily average intakes of energy and nutrients were calculated based on the Chinese Food Composition Tables (6th standard edition), using energy and nutrient contents per 100 grams of edible portion of various foods.

### 2.6 Children's dietary inflammatory index

The C-DII scores in this study were calculated based on dietary data obtained from the 3-day 24-h dietary recall method. Each participant's C-DII score was adapted from the DII developed by Shivappa et al (25). The detailed development and validation of the C-DII have been described in a previous study (26). The present study included 25 dietary components, including energy, carbohydrates, protein, cholesterol, total fat, saturated fat, monounsaturated fatty acids (MUFA), polyunsaturated fatty acids (PUFA), fiber, vitamin A, thiamine, riboflavin, vitamin B6, vitamin B12, vitamin C, vitamin D, vitamin E, niacin, folate, magnesium, iron, zinc, selenium, and β-carotene. Alcohol was not included in the index due to a lack of recorded consumption among participants. Previous studies have demonstrated that the predictive ability of the C-DII for overall dietary inflammatory potential remains consistent when fewer than 30 food parameters are included (25).

In summary, the C-DII was calculated as follows: (1) first, we compared the participants' daily energy and nutrient intakes with the global average intake to calculate the *Z*-scores; (2) we converted *Z*-scores to percentile values, then centralized them by doubling the percentile values and subtracting one; (3) we multiplied the centered values by the corresponding dietary component's inflammation effect score to obtain the individual's inflammation effect scores for each dietary component; (4) Finally, we summed all inflammation scores to obtain each individual's total C-DII score. Higher scores indicate a stronger pro-inflammatory property of the dietary pattern. According to the tertiles of C-DII scores, participants were categorized into three groups: T1 (low C-DII), T2 (medium C-DII), and T3 (high C-DII).

### 2.7 Obstructive sleep apnea 18-item quality-of-life questionnaire (OSA-18)

The OSA-18 was used to assess quality of life (QoL) (42, 43). This instrument is widely used in children with OSAHS. It covers five domains: sleep disturbance, physical symptoms, emotional distress, daytime function, and caregiver concern. The questionnaire comprises 18 items, each scored from one to seven. The total scores are the sum of all item scores, with higher scores indicating a greater impact of OSAHS on the child's quality of life. In this study, the Cronbach's alpha value for the OSA-18 was 0.893.

### 2.8 Covariates

This study included demographic and clinical outcome measures as covariates, including age, sex, current place of residence, only-child status, intake of vitamin D, caregiver's role, caregiver's educational level, caregiver's employment status, parental educational level, average family income, BMI, and A/N ratio.

### 2.9 Statistical analysis

In the descriptive statistics, continuous variables were reported as mean ± standard deviation (SD), while categorical variables were reported as frequency (percentage). For continuous variables, one-way analysis of variance (ANOVA) was used to compare differences across the C-DII tertile groups when the assumption of homogeneity of variance was met, followed by *post-hoc* comparisons using the Bonferroni method. When the homogeneity of variance assumption was violated, Welch's ANOVA was applied, with Tamhane's T2 test used for *post-hoc* comparisons. Comparisons between groups for categorical variables were performed using the chi-square test. If the expected frequency in any cell was less than five, Fisher's exact test was employed instead. Additionally, a chi-square test for trend examined the linear association between C-DII tertiles and the presence of severe OSAHS.

Multiple linear regression analysis was employed to examine the association between C-DII and OAHI. Using the T1 (low C-DII) group as the reference, three models were sequentially constructed: Model 1 adjusted for no variables; Model 2 adjusted for age, sex, current place of residence, only-child status, intake of vitamin D, caregiver's role, caregiver's educational level, caregiver's employment status, parental educational level, average family income, and A/N ratio; Model 3 further adjusted for BMI based on Model 2.

**Figure 2:**
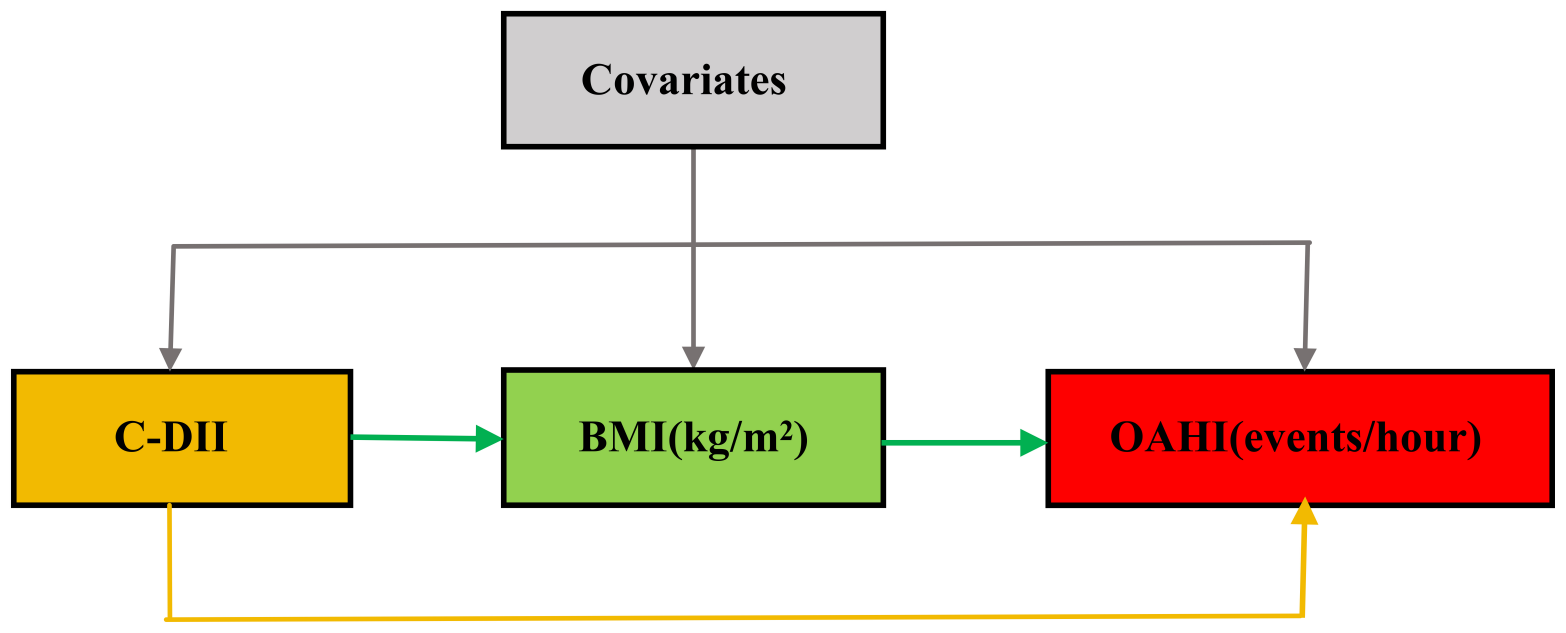
Directed acyclic graph (DAG) of the hypothesized relationships among C-DII, BMI, and OAHI. C-DII, children's dietary inflammatory; BMI, body mass index; OAHI, obstructive apnea-hypopnea index. The yellow box indicates the exposure variable (C-DII). The green box indicates the statistical intermediary (BMI). The red box indicates the outcome variable (OAHI). The gray box indicates covariates included in adjusted models. The yellow path indicates the direct effect and the green paths indicate the indirect effects.

The conceptual framework guiding the mediation analysis is presented as a directed acyclic graph (DAG) in Figure 2. To assess the statistical role of BMI in the association between C-DII and OAHI, we conducted a mediation analysis using the PROCESS macro in SPSS version 26.0 software. This analysis was adjusted for demographic and clinical outcome measures as confounding factors. The 95% confidence interval (CI) for the mediation effect was estimated using 5,000 bootstrap samples.

Stratified analyses were conducted by BMI category (normal weight, overweight, and obesity) according to the BMI cut-off criteria for Chinese children. Within each stratum, linear regression estimated the C-DII–OAHI association adjusting for the same covariates (excluding BMI). All statistical analyses were performed using SPSS version 26.0 software (IBM, Chicago, IL, USA). A two-tailed *p* value < 0.05 was considered statistically significant.

## 3 Results

### 3.1 Characteristics of participants

**Figure 3:**
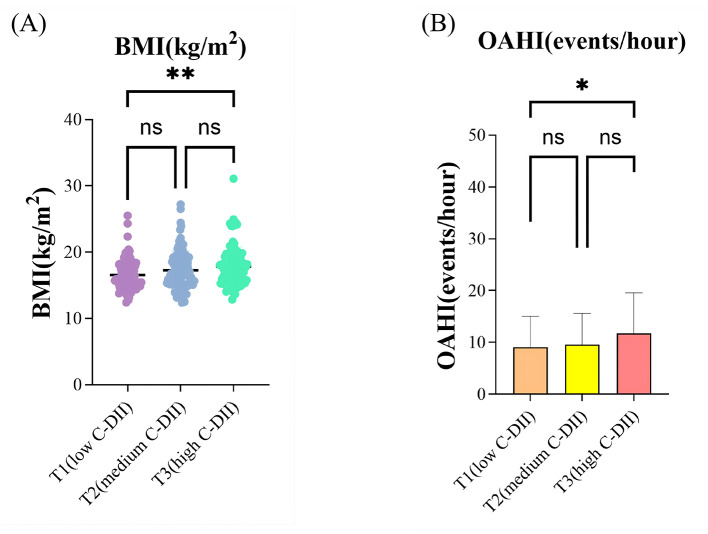
Comparisons of BMI and OAHI among C-DII tertile groups in children with OSAHS. BMI, body mass index; OAHI, obstructive apnea-hypopnea index; C-DII, children's dietary inflammatory index. **(A)** Comparison of BMI among C-DII tertile groups. One-way analysis of variance (ANOVA) with Bonferroni *post-hoc* test was used. **(B)** Comparison of OAHI values among C-DII tertile groups. Welch's ANOVA with Tamhane's T2 *post-hoc* test was used.

This study ultimately included 297 participants, comprising 179 males (60.3%) and 119 females (39.7%). The mean age of participants was 7.54 ± 2.18 years, with an average BMI of 17.21 ± 2.77 kg/m^2^. The mean OAHI was 10.14 ± 6.73 events/h, and C-DII scores ranged from −3.20 to 3.19. Participants were further divided into three groups based on C-DII tertiles: T1 (low C-DII), T2 (medium C-DII), and T3 (high C-DII). Table 1 shows participant characteristics stratified by C-DII tertiles. The mean BMI values of the three groups were 16.53 ± 2.24 kg/m^2^, 17.25 ± 2.93 kg/m^2^, and 17.83 ± 2.96 kg/m^2^, respectively. Significant differences in BMI distribution were observed between groups (*p* = 0.004). *Post-hoc* analysis revealed that the mean BMI level in the T3 (high C-DII) group was significantly higher than that in the T1 (low C-DII) group (*p* = 0.003; Figure 3A).

**Table 1:** Sociodemographic characteristics across C-DII tertiles in children with OSAHS.

Variables	Total (*n* = 297)	T1 (*n* = 99)	T2 (*n* = 99)	T3 (*n* = 99)	*p*-value
Age (years), mean (SD)	7.54 (2.18)	7.16 (2.42)	7.56 (2.17)	7.89 (1.88)	0.063^a^
Sex (*n*, %)					
Female	118 (39.7)	37 (37.4)	32 (32.3)	49 (49.5)	0.040^b^
Male	179 (60.3)	62 (62.6)	67 (67.7)	50 (50.5)
BMI (kg/m^2^), mean (SD)	17.21 (2.77)	16.53 (2.24)	17.25 (2.93)	17.83 (2.96)	0.004^a^
Current place of residence (*n*, %)					
Urban	266 (89.6)	90 (90.9)	90 (90.0)	86 (86.9)	0.592^b^
Rural	31 (10.4)	9 (9.1)	9 (9.1)	13 (13.1)	
Only-child status (*n*, %)					
Yes	141 (47.5)	54 (54.5)	45 (45.5)	42 (42.4)	0.214^b^
No	156 (52.5)	45 (45.5)	54 (54.5)	57 (57.6)	
Vitamin D intake (*n*, %)					
Yes	118 (39.7)	40 (40.4)	38 (38.4)	40 (40.4)	0.966^b^
No	179 (60.3)	59 (59.6)	61 (61.6)	59 (59.6)	
Caregiver's role (*n*, %)					
Parents	203 (68.4)	68 (68.7)	71 (71.7)	64 (64.6)	0.378^b^
Paternal grandparents	57 (19.2)	16 (16.2)	16 (16.2)	25 (25.3)	
Maternal grandparents	37 (12.4)	15 (15.1)	12 (12.1)	10 (10.1)
Caregiver's education level (*n*, %)					
Junior high school or below	127 (42.8)	51 (51.5)	40 (40.4)	36 (36.4)	0.130^b^
Senior high school	42 (14.1)	15 (15.2)	15 (15.2)	12 (12.1)	
University degree or above	128 (43.1)	33 (33.3)	44 (44.4)	51 (51.5)
Caregiver's employment status (*n*, %)					
Full-time	147 (49.5)	46 (46.5)	45 (45.4)	56 (56.6)	0.489^b^
Part-time	44 (14.8)	17 (17.2)	16 (16.2)	11 (11.1)	
Unemployed	106 (35.7)	36 (36.3)	38 (38.4)	32 (32.3)
Father's education level (*n*, %)					
Junior high school or below	66 (22.2)	31 (31.3)	19 (19.2)	16 (16.2)	0.067^b^
Senior high school	78 (26.3)	27 (27.3)	25 (25.2)	26 (26.2)	
University degree or above	153 (51.5)	41 (41.4)	55 (55.6)	57 (57.6)
Mother's education level (*n*, %)					
Junior high school or below	72 (24.3)	21 (21.2)	24 (24.2)	27 (27.3)	0.054^b^
Senior high school	80 (26.9)	18 (18.2)	31 (31.3)	31 (31.3)	
University degree or above	145 (48.8)	60 (60.6)	44 (44.5)	41 (41.4)
Average family income^c^ (*n*, %)					
≤ 5,000	56 (18.9)	23 (23.2)	14 (14.1)	19 (19.2)	0.542^b^
5,001–10,000	131 (44.1)	39 (39.4)	48 (48.5)	44 (44.4)	
>10,000	110 (37.0)	37 (37.4)	37 (37.4)	36 (36.4)

^a^For continuous variables with homogeneity of variance, one-way ANOVA with Bonferroni *post-hoc* test.

^b^For categorical variables, chi-square test.

^c^Yuan/month, 1 yuan = 0.14 dollars.

T1, T1 (low C-DII) group; T2, T2 (medium C-DII) group; T3, T3 (high C-DII) group; BMI, body mass index; C-DII, children's dietary inflammatory index.

Clinical outcome measures stratified by C-DII tertiles are presented in Table 2. Results showed a statistically significant difference in OAHI among the three C-DII groups (*p* = 0.023). *Post-hoc* analysis with Tamhane's T2 test indicated that OAHI was significantly higher in the T3 (high C-DII) group than in the T1 (low C-DII) group (*p* = 0.022), while no significant difference was observed between the T3 (high C-DII) and T2 (medium C-DII) groups (*p* > 0.05; Figure 3B). Furthermore, there were significant differences in quality of life (QoL) scores among children with OSAHS across the C-DII tertiles (*p* = 0.012; Table 2). A significant positive linear trend was observed between C-DII tertiles and the presence of severe OSAHS (χ^2^ = 7.287, *p* = 0.007), indicating that the proportion of severe cases increased with higher tertile levels.

**Table 2:** Clinical outcome measures of children with OSAHS across tertiles of the C-DII.

Variables	Total (*n* = 297)	T1 (*n* = 99)	T2 (*n* = 99)	T3 (*n* = 99)	*p*-value
OAHI	10.14 (6.73)	9.07 (6.00)	9.60 (6.00)	11.75 (7.80)	0.023^a^
A/N ratio	0.74 (0.05)	0.73 (0.03)	0.74 (0.05)	0.75 (0.07)	0.009^a^
OSA-18	70.96 (17.79)	69.11 (19.72)	68.70 (17.12)	75.07 (15.77)	0.012^a^
Sleep disturbance	14.84 (5.84)	14.90 (6.29)	13.80 (5.30)	15.83 (5.76)	0.049^b^
Physical symptoms	16.83 (4.74)	16.67 (4.86)	16.44 (4.82)	17.37 (4.53)	0.355^b^
Emotional distress	10.77 (4.24)	10.15 (4.61)	10.69 (4.08)	11.47 (3.94)	0.087^b^
Daytime function	9.02 (3.77)	8.86 (3.62)	8.72 (3.63)	9.47 (4.05)	0.324^b^
Caregiver concern	19.50 (6.32)	18.54 (6.92)	19.05 (5.94)	20.92 (5.85)	0.018^a^

^a^For continuous variables with heterogeneity of variance, Welch's ANOVA with Tamhane's T2 *post-hoc* test.

^b^For continuous variables with homogeneity of variance, one-way ANOVA with Bonferroni *post-hoc* test.

C-DII, children's dietary inflammatory index; T1, T1 (low C-DII) group; T2, T2 (medium C-DII) group; T3, T3 (high C-DII) group; OAHI, obstructive apnea-hypopnea index; A/N, adenoidectomy/nasopharyngeal ratio; OSA-18, obstructive sleep apnea 18-item quality-of-life questionnaire. Continuous variables are presented as mean (standard deviation).

### 3.2 Dietary intakes across C-DII tertiles

Supplementary Table 2 presents comparisons of energy and nutrient intakes across the three C-DII tertile groups. The results showed significant differences among the groups in the intakes of energy, carbohydrates, protein, total saturated fat, MUFA, PUFA, and fiber (all *p* < 0.05). For the remaining nutrients, no significant differences were observed among the three groups (all *p* > 0.05).

### 3.3 Association between C-DII scores and OAHI

To further explore the association between different C-DII levels and OAHI, we conducted multiple linear regression analysis with the T1 (low C-DII) group as the reference, with results shown in Table 3. In the unadjusted Model 1, the T3 (high C-DII) group showed a significant increase in OAHI of 2.68 events/h compared to the T1 (low C-DII) group (β = 2.68, 95% CI: 0.82–4.54, *p* = 0.005). In Model 2 (partially adjusted model), the T3 (high C-DII) group still showed a significantly higher OAHI compared to the T1 (low C-DII) group (β = 2.04, 95% CI: 0.07–4.01, *p* = 0.042). However, when BMI was further incorporated into Model 2 (Model 3), the OAHI difference between the T3 and T1 groups was no longer statistically significant (β = 1.58, 95% CI: −0.37–3.54, *p* = 0.112). Meanwhile, BMI showed a significant positive association with OAHI (β = 0.53, 95% CI: 0.22–0.85, *p* = 0.001).

**Table 3:** Multiple linear regression analysis for C-DII scores and OAHI.

Variables	Model 1		Model 2		Model 3	
	β (95% CI)	*p*-value	β (95% CI)	*p*-value	β (95% CI)	*p-*value
T1 (low C-DII)	reference		reference		reference	
T2 (medium C-DII)	0.53 (−1.33–2.39)	0.578	−0.10 (−2.01–1.19)	0.920	−3.50 (−2.24–1.54)	0.715
T3 (high C-DII)	2.68 (0.82–4.54)	0.005	2.04 (0.07–4.01)	0.042	1.58 (−0.37–3.54)	0.112
BMI	–		–		0.53 (0.22–0.85)	0.001
*R* ^2^	0.030		0.086		0.120	
Δ*R*^2^	0.023		0.040		0.073	
*F*	4.504		1.884		2.552	
*p*-value	0.012		0.028		0.001	

Model 1: no covariates were adjusted; Model 2: adjusted for age, sex, current place of residence, only-child status, intake of vitamin D, caregiver's role, caregiver's educational level, caregiver's employment status, parental educational level, average family income, and A/N ratio. Model 3: additionally adjusted for BMI based on Model 2.

CI, confidence interval; C-DII, children's dietary inflammatory index; OAHI, obstructive apnea-hypopnea index; BMI, body mass index.

### 3.4 Mediation analysis of BMI on association of C-DII with OAHI

**Figure 4:**
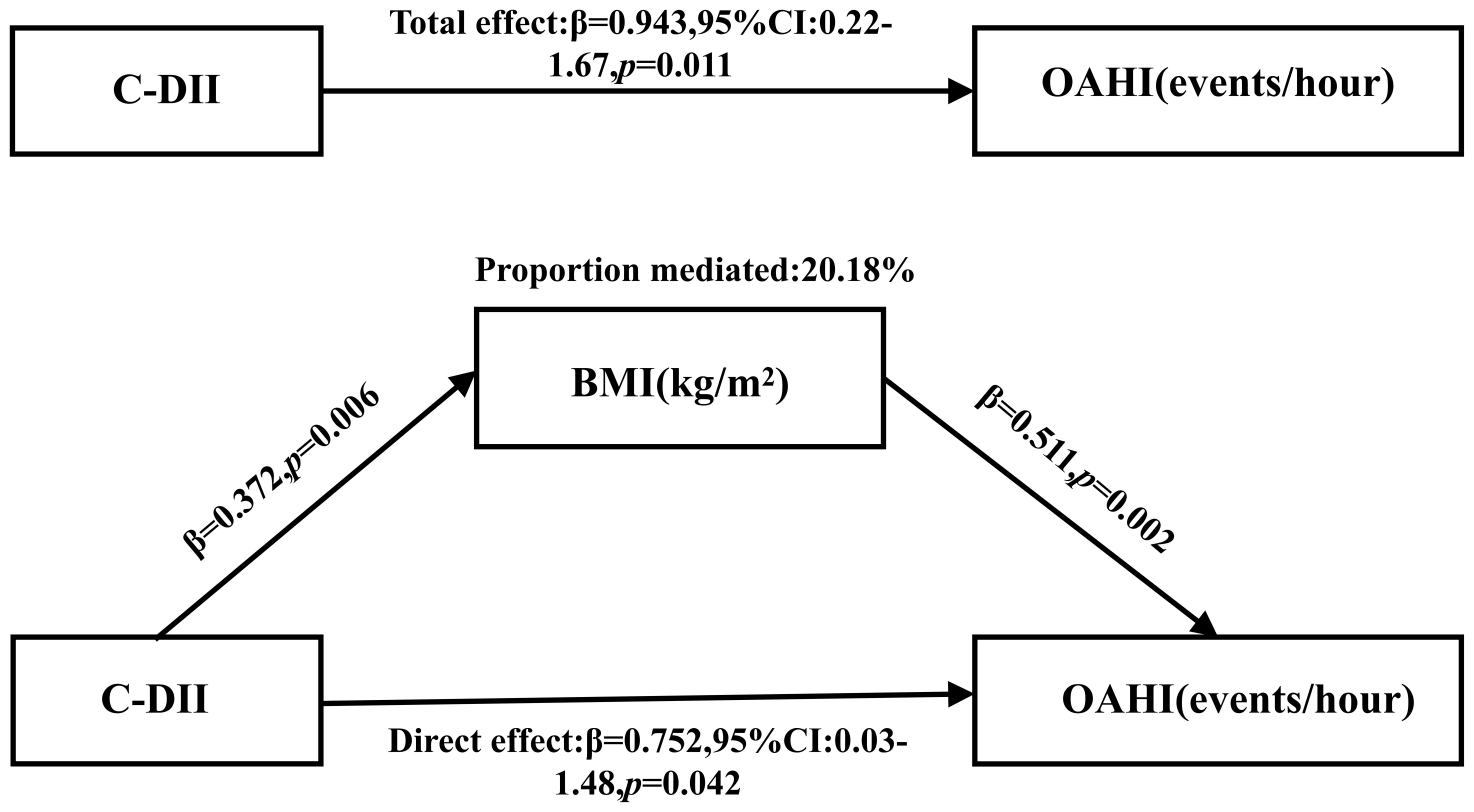
Mediating effect of BMI in the association between the C-DII and OAHI. BMI, body mass index; C-DII, children's dietary inflammatory index; OAHI, obstructive apnea-hypopnea index. Adjusted for age, sex, current place of residence, only-child status, intake of vitamin D, caregiver's role, caregiver's educational level, caregiver's employment status, parental educational level, average family income, and A/N ratio.

As shown in Figure 4, a statistical mediation model was fitted to quantify the indirect association between C-DII and OAHI through BMI. After adjusting for all covariates, the results indicated that the total statistical association between C-DII and OAHI was 0.94 (95% CI: 0.22–1.67, *p* = 0.011). The direct association (independent of BMI) was 0.75 (95% CI: 0.03–1.48, *p* = 0.0416). The indirect association statistically accounted for by BMI was 0.19, representing 20.18% of the total observed association between C-DII and OAHI see Supplementary Table 3.

### 3.5 Stratified analysis by BMI categories

To assess whether the association between C-DII and OAHI differed by weight status, we performed linear regression analyses stratified by BMI. After covariate adjustment, C-DII score was not associated with OAHI in the normal weight group (β = 0.097, 95% CI: −0.746–0.940, *p* = 0.822), the overweight group (β = 0.902, 95% CI: −1.626–3.430, *p* = 0.488), or the obesity group (β = −0.944, 95% CI: −3.908–2.020, *p* = 0.538) see Table 4.

**Table 4:** Stratified analysis of the association between C-DII and OAHI by BMI categories.

BMI category	*n* (%)	β (95% CI)	*R* ^2^	Δ*R*^2^	*p*-value
Normal weight	196 (65.99)	0.097 (−0.746–0.940)	0.051	−0.016	0.822
Overweight	61 (20.54)	0.902 (−1.626–3.430)	0.219	0.003	0.488
Obesity	40 (13.47)	−0.944 (−3.907–2.019)	0.458	0.187	0.538

BMI, body mass index; CI, confidence interval; Models were adjusted for age, sex, current place of residence, only-child status, intake of vitamin D, caregiver's role, caregiver's educational level, caregiver's employment status, parental educational level, average family income, and A/N ratio.

## 4 Discussion

In this cross-sectional study, after controlling for confounding factors, a high C-DII level was significantly associated with higher OAHI in children with OSAHS compared with a low C-DII level. Crucially, our findings provide novel statistical evidence that BMI statistically accounts for a portion of this association, representing approximately 20.18% of the total observed association. The statistical association between C-DII and OAHI was attenuated and no longer statistically significant after additionally adjusting for BMI, while BMI itself remained a strong, independent correlate of OAHI. This pattern indicates that the observed association between dietary inflammation and OSAHS severity is largely explained by BMI.

Our results are consistent with the well-documented association between pro-inflammatory diets and elevated BMI in children (27–29). After stratification by C-DII tertiles, children in the highest tertile (T3) had a significantly higher BMI than those in the lowest tertile (T1). This group exhibited a dietary pattern characterized by higher energy intake, higher saturated fat intake, and lower fiber intake—a profile consistently linked to weight gain (44). In children with OSAHS, these associations may be further reinforced. A pro-inflammatory diet (i.e., high C-DII scores) has been hypothesized to disrupt gut microbiota homeostasis and weaken intestinal barrier function, thereby promoting a systemic low-grade inflammatory response (45). This systemic inflammatory state may not only coincide with the inherent chronic inflammatory response of OSAHS but also impair children's energy regulation, potentially contributing to higher BMI in OSAHS patients with elevated C-DII.

The pro-inflammatory dietary pattern reflected by high C-DII scores is characterized by chronic inflammatory stimulation. This study found that after adjusting for sociodemographic and disease-related covariates (excluding BMI), the association between a high C-DII and worse OAHI remained significant, suggesting the existence of BMI-independent statistical associations. Diets high in inflammatory potential are typically rich in refined carbohydrates, added sugars, and ultra-processed foods, while being deficient in anti-inflammatory components found in fruits, vegetables, and whole grains (46, 47). Such diets may exacerbate OSAHS through mechanisms that include promoting systemic inflammation, which can worsen upper airway mucosal edema and impair neuromuscular control of the airway, thereby increasing its collapsibility during sleep (32, 48). This interpretation aligns with adult studies in which higher DII scores were associated with increased AHI and daytime sleepiness, independent of weight (36, 49, 50). Nevertheless, in our pediatric participants, the independent contribution of dietary inflammation was minimal after adjusting for BMI, suggesting that the direct inflammatory effects of diet on the upper airway may be less pronounced in children than in adults or may require a longer exposure period to manifest.

A central finding of this study is the statistical role of BMI in the diet-OSAHS relationship. The stratified regression analysis revealed that additional adjustment for BMI rendered the direct C-DII-OAHI statistical association non-significant, underscoring the strong overlap between dietary inflammation and adiposity in relation to OSAHS severity. The subsequent mediation analysis quantified that BMI statistically accounted for approximately one-fifth of the total observed association. This pattern is consistent with the biological understanding that a pro-inflammatory diet promotes weight gain, and elevated BMI, in turn, is a well-established correlate of OSAHS severity. Mechanistically, obesity may contribute to OSAHS through both anatomical compression of the pharyngeal airway by excess neck fat (14, 15, 51) and the release of pro-inflammatory cytokines from adipose tissue, which can exacerbate local inflammation in the upper airway (52–54). A pro-inflammatory diet may promote weight gain. Increased adiposity, in turn, is associated with worsened airway obstruction, potentially perpetuating a self-reinforcing cycle. This mediation approach also mitigates concerns regarding overadjustment bias that could arise if BMI were treated simply as a covariate in the primary regression models.

With childhood obesity and pediatric OSAHS both on the rise, weight management remains a cornerstone of non-surgical intervention. Our findings suggest that anti-inflammatory dietary patterns may benefit children with OSAHS primarily by facilitating weight control rather than by directly ameliorating upper airway inflammation. This does not diminish the importance of dietary quality. On the contrary, it clarifies that interventions aimed at reducing intake of saturated fats and refined sugars, while increasing consumption of anti-inflammatory foods including dietary fiber, fruits, vegetables, and whole grains, may help mitigate OSAHS severity by preventing excessive weight gain (55–57). Adopting anti-inflammatory dietary patterns, such as the Mediterranean diet, has shown promise in improving AHI and inflammatory markers in adults with OSAHS (58, 59), and our study provides a strong rationale for testing similar interventions in pediatric populations. From a public health perspective, these findings support the potential value of school-based nutrition education and community initiatives aimed at reducing dietary inflammation and promoting healthy weight as strategies to help mitigate the burden of pediatric OSAHS. Future studies should evaluate the feasibility and effectiveness of implementing such programs in diverse settings.

This study has several strengths, including its focus on a pediatric OSAHS population, the use of a validated tool (C-DII) to assess dietary inflammation, the application of rigorous statistical models including mediation analysis, and the adjustment for key clinical confounders like the A/N ratio. However, several limitations should be acknowledged. First, the cross-sectional design precludes causal inferences. Reverse causality, such as OSAHS affecting dietary behaviors or physical activity and thereby BMI, cannot be excluded, and the temporal sequence among diet, adiposity, and OSAHS severity remains uncertain. These findings should therefore be regarded as hypothesis-generating. Second, dietary data from caregiver-reported 24-h recalls are subject to recall bias, although we included both weekdays and a weekend day and used standardized aids to improve accuracy. Although food frequency questionnaires may offer more stable estimates of habitual intake, the 3-day recall was selected for its ability to capture detailed recent nutrient data relevant to current OSAHS severity. Third, residual confounding from unmeasured factors including physical activity, sleep duration, pubertal status, and environmental exposures may persist. Finally, the single-center convenience sample may limit the generalizability. Caution is therefore warranted when generalizing these findings to other pediatric populations. Future prospective studies and multicenter trials are needed to confirm these findings and evaluate the efficacy of anti-inflammatory dietary interventions in pediatric OSAHS.

## 5 Conclusion

In conclusion, this cross-sectional study demonstrated that a more pro-inflammatory diet, as reflected by a higher C-DII score, was associated with greater OSAHS severity in children. In statistical terms, BMI accounted for approximately one-fifth of this observed association. Given the cross-sectional design, causality cannot be inferred, and the independent contribution of dietary inflammation beyond BMI remains uncertain. These findings highlight the potential value of integrating dietary improvement with weight management as part of a comprehensive approach to pediatric OSAHS care. Future longitudinal studies are warranted to clarify whether specific anti-inflammatory dietary interventions confer benefits independent of weight control.

## Data Availability

The original contributions presented in the study are included in the article/Supplementary material, further inquiries can be directed to the corresponding author/s.
