# Supplementary material for: Body mass index mediates the association between children's dietary inflammatory index and obstructive sleep apnea-hypopnea syndrome in children: a cross-sectional study
Source: Front Public Health. 2026 Jun 5;14:1772032. doi: 10.3389/fpubh.2026.1772032 (PMC13279400; doi:10.3389/fpubh.2026.1772032)
Supplement: Supplementary file 1 [file Table_1.docx]

Supplementary Material

**Table S1.** STROBE Statement—Checklist of items that should be included in reports of ***cross-sectional study.***

|  | **Item No** | **Recommendation** | **Page No** |
| --- | --- | --- | --- |
| **Title and abstract** | 1 | (*a*) Indicate the study’s design with a commonly used term in the title or the abstract | Title |
|  |  | (*b*) Provide in the abstract an informative and balanced summary of what was done and what was found | Abstract |
| **Introduction** | | | |
| Background/rationale | 2 | Explain the scientific background and rationale for the investigation being reported | 1.Introduction |
| Objectives | 3 | State specific objectives, including any prespecified hypotheses | 1.Introduction |
| **Methods** | | | |
| Study design | 4 | Present key elements of study design early in the paper | 2.1 Study design |
| Setting | 5 | Describe the setting, locations, and relevant dates, including periods of recruitment, exposure, follow-up, and data collection | 2.Materials and methods |
| Participants | 6 | (*a*) Give the eligibility criteria, and the sources and methods of selection of participants | 2.2 Participants |
|  |  | (*b*) For matched studies, give matching criteria and number of exposed and unexposed | Not applicable |
| Variables | 7 | Clearly define all outcomes, exposures, predictors, potential confounders, and effect modifiers. Give diagnostic criteria, if applicable | 2.Materials and methods |
| Data sources/ measurement | 8* | For each variable of interest, give sources of data and details of methods of assessment (measurement). Describe comparability of assessment methods if there is more than one group | 2.Materials and methods |
| Bias | 9 | Describe any efforts to address potential sources of bias | 2.Materials and methods |
| Study size | 10 | Explain how the study size was arrived at | 2.3 Sample size |
| Quantitative variables | 11 | Explain how quantitative variables were handled in the analyses. If applicable, describe which groupings were chosen and why | 2.Materials and methods |
| Statistical methods | 12 | (*a*) Describe all statistical methods, including those used to control for confounding | 2.Materials and methods |
|  |  | (*b*) Describe any methods used to examine subgroups and interactions | 2.Materials and methods |
|  |  | (*c*) Explain how missing data were addressed | None |
|  |  | (*d*) If applicable, describe analytical methods taking account of sampling strategy | 2.Materials and methods |
|  |  | (*e*) Describe any sensitivity analyses | None |
| **Results** | | |  |
| Participants | 13* | (a) Report numbers of individuals at each stage of study—eg numbers potentially eligible, examined for eligibility, confirmed eligible, included in the study, completing follow-up, and analysed | 3.1 Characteristics of participants |
|  |  | (b) Give reasons for non-participation at each stage | 2.Materials and methods |
|  |  | (c) Consider use of a flow diagram | 2.Materials and methods |
| Descriptive data | 14* | (a) Give characteristics of study participants (eg demographic, clinical, social) and information on exposures and potential confounders | 3. Results |
|  |  | (b) Indicate number of participants with missing data for each variable of interest | 3. Results |
|  |  | (c) Summarise follow-up time (eg, average and total amount) | Not applicable |
| Outcome data | 15* | Report numbers of outcome events or summary measures over time | 3. Results |

| Main results | 16 | (*a*) Give unadjusted estimates and, if applicable, confounder-adjusted estimates and their precision (eg, 95% confidence interval). Make clear which confounders were adjusted for and why they were included | 3. Results |
| --- | --- | --- | --- |
|  |  | (*b*) Report category boundaries when continuous variables were categorized | 3. Results |
|  |  | (*c*) If relevant, consider translating estimates of relative risk into absolute risk for a meaningful time period | Not applicable |
| Other analyses | 17 | Report other analyses done—eg analyses of subgroups and interactions, and sensitivity analyses | None |
| **Discussion** | | | |
| Key results | 18 | Summarise key results with reference to study objectives | 4.Discussion |
| Limitations | 19 | Discuss limitations of the study, taking into account sources of potential bias or imprecision. Discuss both direction and magnitude of any potential bias | 4.Discussion |
| Interpretation | 20 | Give a cautious overall interpretation of results considering objectives, limitations, multiplicity of analyses, results from similar studies, and other relevant evidence | 4.Discussion |
| Generalisability | 21 | Discuss the generalisability (external validity) of the study results | 4.Discussion |
| **Other information** | | | |
| Funding | 22 | Give the source of funding and the role of the funders for the present study and, if applicable, for the original study on which the present article is based | 8.Funding |

*Give information separately for T1 (low C-DII), T2 (medium C-DII), and T3 (high C-DII) groups.

**Table S2.** Dietary intake of children with OSAHS across tertiles of the C-DII.

| Variables | Total(n=297) | T1 (n=99) | T2(n=99) | T3(n=99) | *p*-value |
| --- | --- | --- | --- | --- | --- |
| Energy, kcal | 1815.70(253.77) | 1756.34(147.77) | 1779.49(167.96) | 1911.28(361.06) | 0.001^a^ |
| Carbohydrate, g | 249.86(28.54) | 244.18(14.02) | 246.27(23.40) | 259.12(39.80) | 0.002^a^ |
| Protein, g | 53.61(10.87) | 49.54(7.16) | 51.75(8.00) | 59.56(13.62) | ˂0.001^a^ |
| Cholesterol, mg | 259.23(46.25) | 252.37(23.25) | 258.31(42.01) | 266.70(63.61) | 0.071^a^ |
| Total fat, g | 65.07(33.11) | 61.73(6.93) | 63.33(7.93) | 70.14(56.22) | 0.165^b^ |
| Total saturated fat, g | 20.95(4.94) | 19.63(4.49) | 21.36(5.01) | 21.88(5.07) | 0.002^a^ |
| MUFA, g | 23.02(5.48) | 23.97(5.74) | 23.10(4.97) | 21.99(5.57) | 0.038^b^ |
| PUFA, g | 14.28(4.03) | 15.86(4.47) | 13.77(3.70) | 13.20(3.37) | ˂0.001^a^ |
| Fiber, g | 13.61(2.98) | 14.55(3.31) | 13.11(2.64) | 13.18(2.76) | 0.002^a^ |
| Vitamin A, mcg | 497.42(111.43) | 513.97(116.02) | 505.05(113.50) | 473.24(101.15) | 0.025^b^ |
| Thiamin, mg | 1.00(0.30) | 0.98(0.29) | 0.96(0.22) | 1.07(0.36) | 0.030^a^ |
| Riboflavin, mg | 0.98(0.30) | 1.02(0.31) | 0.97(0.25) | 0.96(0.32) | 0.334^b^ |
| Vitamin B6, mg | 1.09(0.31) | 1.13(0.35) | 1.04(0.21) | 1.08(0.33) | 0.092^a^ |
| Vitamin B12, mcg | 2.08(0.65) | 2.19(0.65) | 2.13(0.61) | 1.93(0.67) | 0.017 ^a^ |
| Vitamin C, mg | 93.41(18.91) | 108.27(16.09) | 86.66(15.20) | 85.30(15.97) | ˂0.001^b^ |
| Vitamin D, mcg | 7.29(3.15) | 9.55(2.95) | 7.33(2.73) | 4.98(1.79) | ˂0.001^a^ |
| Vitamin E, mg | 12.49(4.16) | 14.44(3.23) | 13.38(3.91) | 9.64(3.70) | ˂0.001^b^ |
| Niacin, mg | 15.23(4.11) | 15.88(3.45) | 16.16(3.87) | 13.66(4.50) | ˂0.001^b^ |
| Folate, mcg | 312.66(76.81) | 353.27(65.05) | 316.91(69.12) | 267.78(71.35) | ˂0.001^b^ |
| Magnesium, mg | 279.97(76.25) | 322.17(57.21) | 289.95(65.72) | 227.80(72.94) | ˂0.001^b^ |
| Iron, mg | 14.02(1.91) | 14.17(0.82) | 14.26(1.41) | 13.63(2.84) | 0.143^a^ |
| Zinc, mg | 10.48(1.13) | 10.71(0.55) | 10.72(0.91) | 10.00(1.53) | ˂0.001^a^ |
| Selenium, mcg | 60.55(31.21) | 85.51(26.10) | 60.95(23.93) | 32.20(20.23) | ˂0.001^b^ |
| β-carotene, mcg | 2560.01(1337.85) | 3531.80(1332.14) | 2252.57(1295.37) | 1895.65(676.85) | ˂0.001^a^ |

^a^ For continuous variables with heterogeneity of variance, Welch’s ANOVA with Tamhane’s T2 post-hoc test; ^b^ For continuous variables with homogeneity of variance, one-way ANOVA with Bonferroni post-hoc test; T1, T1 (low C-DII) group; T2, T2 (medium C-DII) group; T3, T3 (high C-DII) group; MUFA, monounsaturated fatty acids; PUFA, polyunsaturated fatty acids. Continuous variables are presented as mean (standard deviation).

**Table S3.** Mediating effect of BMI in the association between the C-DII and OAHI.

| Variables | β (95% CI) | *p*-value |
| --- | --- | --- |
| Mediator: BMI |  |  |
| Total effect | 0.94(0.22-1.67) | 0.011 |
| Direct effect | 0.75(0.03-1.48) | 0.0416 |
| Mediation effect | 0.19(0.06-0.37) | - |
| Proportion mediated, % | 20.18% | - |

CI, confidence intervals; C-DII, children's Dietary Inflammatory Index; BMI, body mass index. Adjusted for age, sex, current place of residence, only-child status, intake of vitamin D, caregiver's role, caregiver's educational level, caregiver's employment status, parental educational level, average family income, and A/N ratio.
